# Severe immune dysregulation with neurological impairment and minor bone changes in a child with spondyloenchondrodysplasia due to two novel mutations in the ACP5 gene

**DOI:** 10.1186/s12969-015-0035-7

**Published:** 2015-09-07

**Authors:** Hermann Girschick, Christine Wolf, Henner Morbach, Christoph Hertzberg, Min Ae Lee-Kirsch

**Affiliations:** Children’s Hospital, Vivantes Hospital im Friedrichshain, Berlin, Germany; Department of Pediatrics, Medizinische Fakultät Carl Gustav Carus, Technische Universität Dresden, Dresden, Germany; Department of Pediatrics, University Clinics of Würzburg, Würzburg, Germany; Socialpediatric Centre, Children’s Hospital, Vivantes Hospital Neukölln, Berlin, Germany

**Keywords:** Spondyloenchondrodysplasia, ACP5, TRAP, Autoimmunity, Immunodeficiency, Type I interferonopathy

## Abstract

Spondyloenchondrodysplasia (SPENCD) is a rare skeletal dysplasia, characterized by metaphyseal lesions, neurological impairment and immune dysregulation associated with lupus-like features. SPENCD is caused by biallelic mutations in the *ACP5* gene encoding tartrate-resistant phosphatase. We report on a child, who presented with spasticity, multisystem inflammation, autoimmunity and immunodeficiency with minimal metaphyseal changes due to compound heterozygosity for two novel *ACP5* mutations. These findings extend the phenotypic spectrum of SPENCD and indicate that *ACP5* mutations can cause severe immune dysregulation and neurological impairment even in the absence of metaphyseal dysplasia.

## Background

Spondyloenchondrodysplasia (SPENCD) is a rare skeletal dysplasia, characterized by enchondromatous non-ossifying metaphyseal and spondylar lesions [1, 2]. Patient may exhibit varying degrees of neurological impairment including spasticity, developmental delay and basal ganglia calcification [3]. In addition, signs of autoimmune disease resembling systemic lupus erythematosus (SLE) are commonly observed [2]. Furthermore, patients may also suffer from recurrent infections. SPENCD is inherited in an autosomal recessive manner and was recently shown to be caused by bilallelic mutations in the *ACP5* gene encoding tartrate-resistant phosphatase (TRAP) [4, 5]. It was shown that loss of TRAP activity in patients with SPENCD results in decreased dephosphorylation of osteopontin, a cytokine present in bone-dissolving osteoclasts as well as in antigen-presenting macrophages and dendritic cells [4, 5]. Elevated levels of active phosphorylated osteopontin are thought to be responsible for increased bone resorption and immune dysregulation resulting in skeletal abnormalities and an altered cytokine profile characterized by overproduction of type I interferon.

## Case presentation

**Fig. 1:**
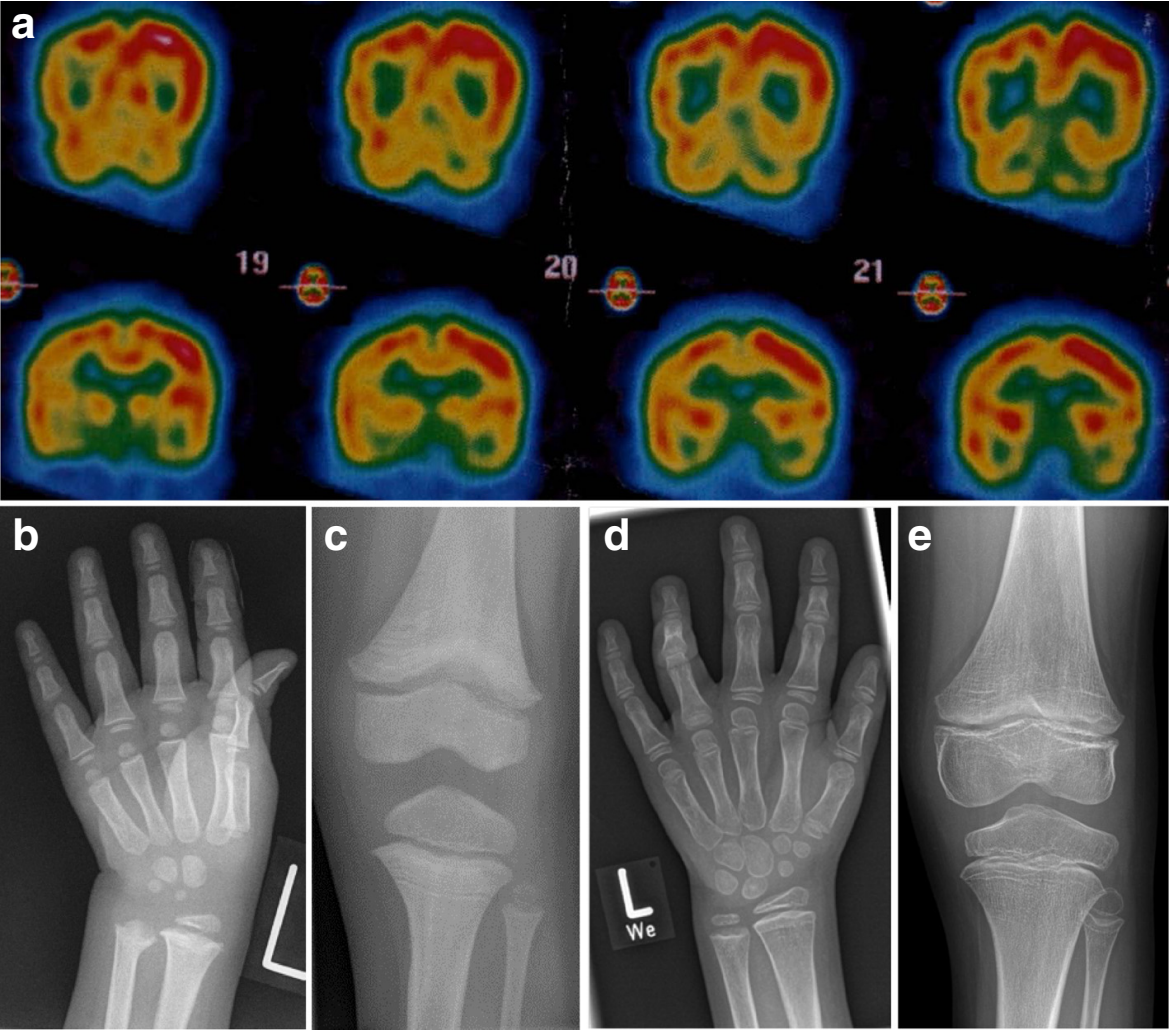
Clinical findings. **a** Single-photon emission computed tomography (SPECT) of the brain at 5 years of age demonstrating speckled areas of reduced perfusion predominantly in the frontal, temporal and parietal regions of the right cortex as well as in parts of the basal ganglia consistent with cerebral vascular lesions. Radiographs of the left hand and knee taken at 4 (**b**, **c**) and 9 years of age (**d**, **e**), respectively, showing discrete metaphyseal irregularities. In addition, reduced mineralization of the lower extremities caused by impaired physical activity is seen at 9 years of age

We report on a 9 year-old Caucasian girl who was born at term to non-consanguineous healthy parents after an uncomplicated pregnancy. Following a period of normal development, an increased muscle tone of the lower limbs was first noted at 6 months of age. An MRI of the brain was normal at this time. Laboratory testing did not reveal any signs of inflammation, lysosomal storage diseases or neurometabolic disease. At 18 months of age she was unable to sit unsupported or to crawl. Hearing and vision were not impaired. Liver function tests were abnormal (ALAT 107 U/l, ASAT 133 U/l) and she tested positive for IgG against EBNA1, VCA-p18, VCA-p23, IEA-BZLF1, EA-p138 and EA-p54 consistent with a past EBV infection. Infections with cytomegalovirus, hepatitis B and C viruses were excluded. Examination of cerebrospinal fluid (CSF) revealed normal values for cell counts, protein, glucose, lactate and neurotransmitters. However, interferon-α was elevated in CSF (6 IU/ml [<2 IU/ml]) as well as in serum (100 IU/ml [<2 IU/ml]) which was attributed to chronic EBV infection. At 2.5 years of age, thrombocytopenia (4/nl) and mild anaemia (haemoglobin 8.8 g/dl) were noted. The patient now tested positive for autoantibodies including ANA (1:1280; speckled pattern), antibodies against Ro/SS-A, La/SS-B, topoisomerase as well as p-ANCA (1:160). Complement levels were reduced (C3 39 mg/dl [80–150 mg/dl]; C4 7.8 mg/dl [12.5–42.5 mg/dl]). An MRI of the brain showed delayed myelinisation, no calcifications were seen on a CT scan. Single-photon emission computed tomography revealed areas of disturbed perfusion in the frontal, temporal and parietal regions of the cortex as well as in the basal ganglia consistent with cerebral vascular disease (Fig. 1a). The patient was started on methylprednisolone pulse therapy every 8 weeks. The parents noted some improvement of spasticity. At 4.5 years of age, elevated transaminases as well as ANA (1:640) and p-ANCA antibodies (1:160) were still detectable. Mycophenolate mofetil (250 mg/day) was started with some improvement of motor function. Two months later, the patient presented with a mucosal bleeding disorder that was attributed to thrombocytopenia (33/nl). The girl was started on prednisolone (5 mg every other day). Mycophenolate was stopped for 6 weeks, during which time platelets further decreased suggesting that thrombocytopenia was not a side effect of this medication. Mycophenolate was therefore restarted with 400 mg daily. Under this combination therapy her platelet count rose to 248/nl.

At 5 years of age the child was hospitalized with high fever, pneumonia and bloody diarrhoea. She tested positive for mycoplasma IgG, but no IgM, and was started on erythromycin. No infectious agents were detected in the stool. Anaemia worsened (Hb 6.0 g/dl) and the girl required a blood transfusion. A positive direct Coombs test confirmed autoimmune haemolytic anaemia. The patient also developed erythema nodosum of the lower limbs and a severe polyarthritis affecting wrist, hip, ankle and knee joints. During the course of this illness, antibodies against β2-glycoprotein IgM and IgG as well as anti-cardiolipin IgG and IgM along with a severely altered coagulation profile due to anti-phospholipid syndrome were noted. Prednisolone was increased to 20 mg daily, which led to a marked improvement of clinical symptoms.

In the following months, the patients experienced recurrent infections affecting the urinary tract and upper airways as well as an episode of severe aphthous stomatitis accompanied by Quinke oedema of the face. In addition, the girl developed recurrent petechiae of the face, erythema nodosum of the feet as well as mild sugillations of the oral mucosa despite normal platelet counts. Prednisolone could not be reduced below 10 mg per day because of recurring polyarthritis. Further immunological work up revealed a reduced number of CD4+ cells (268/μl [700–2200/μl]), B cells (0.2 % [1–4 %]) and natural killer cells (2 % [4–26 %]).

At 5.5 years of age, she presented with a high fever, generalized vasculitis, encephalopathy with seizures and respiratory insufficiency due to interstitial pneumonia requiring 2 days of mechanical ventilation. She also developed polyarthritis and nephritis with proteinuria. Blood cultures were positive for ESBL-positive Klebsiella pneumoniae. Finally, the diagnosis of acute measles infection was made, in addition to reactivated EBV infection. The girl had not been vaccinated with life vaccines in the past due to immunosuppression and in part due to parental refusal. Seizures were treated with phenobarbital and levetiracetam. Antibiotic therapy with cefuroxime and imipenem were started along with methylprednisolone pulse therapy for 3 days. She also received immunoglobulins because of severe hypogammaglobulinaemia.

At 7 years of age, the girl presented with high fever, severe polyarthritis, enthesitis of the wrists and oral mucositis with sugillations. At that time, mumps, herpes simplex stomatitis and adenoviral infection were diagnosed, in addition to EBV reactivation. The patient received intravenous IgG substitution, acyclovir and prednisolone 15 mg per day. In addition, antibiotic therapy using amoxicillin/sulbactam was started and continued at a prophylactic dosage for half a year because of recurrent genital ulcers and abscesses.

**Fig. 2:**
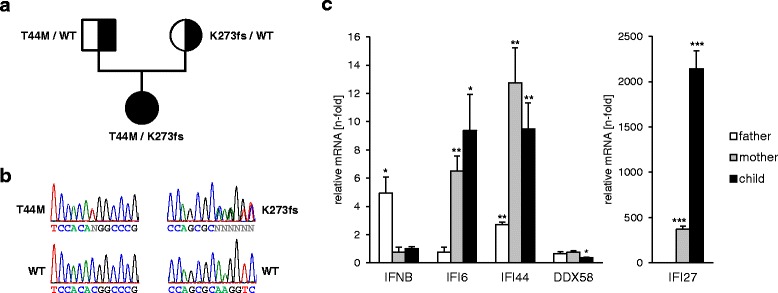
Molecular findings. **a** Pedigree of patient’s family with *ACP5* gene mutations indicated. **b** Pherograms showing *ACP5* gene mutations as sequenced in the patient. **c** Expression of *IFNB* and the interferon-stimulated genes *IFI6*, *IFI44*, *DDX58* and *IFI27* in peripheral blood cells normalized to *GAPDH*. Indicated are the fold changes in gene expression relative to the mean gene expression of 2 wild type controls. Gene expression was determined by quantitative RT-PCR. Shown are the means ± SEM of triplicate measurements. *p < 0.05; **p < 0.01; ***p < 0.001, 2-tailed Student’s t test

As part of her diagnostic work-up, genetic testing for TNF-associated periodic syndrome, Hyper IgD syndrome, familial Mediterranean fever, cryopyrin associated periodic syndrome and Aicardi-Goutières syndrome was performed with negative results. Whole body MRI at that time did not reveal structural changes of the joints including wrists, knees and spine. Because of short stature and discrete metaphyseal changes of the wrist (Fig. 1b), the *ACP5* gene encoding tartrate resistant acid phosphatase (TRAP) was sequenced. The patient was found to be compound heterozygous for a missense mutation (c.131C > T [p.Thr44Met, T44M]) and a frameshift mutation (c.816dupC [p.K272Qfs*14, K272fs]) which was predicted to result in a truncated protein. The T44M mutation was listed in the ENSEMBL database with an allele frequency of 0.0001 and was predicted to be pathogen (SIFT: deleterious; POLYPHEN: probably damaging). Both parents were found to be heterozygous for one of the mutations. Biochemically, TRAP activity was undetectable in the patient, while both parents exhibited markedly decreased TRAP levels (mother 1.5 U/l [<4.3 U/l]; father 2.2 U/l [<5.4 U/l]) confirming the diagnosis of SPENCD. The patient was found to exhibit up-regulation of interferon-stimulated genes in peripheral blood (Fig. 2). Interestingly, both clinically healthy parents also showed an interferon signature in peripheral blood (Fig. 2). Antibodies to nuclear antigens were detected in the mother’s blood.

## Discussion

We describe an unusual case of SPENCD due to two hitherto unknown mutations in the *ACP5* gene leading to complete loss of TRAP activity in the patient’s serum. The clinical picture of the patient reported here is characterized by neurological impairment due to cerebral vascular disease as well as severe immune dysregulation resulting in autoimmunity and immunodeficiency. Thus, in addition to autoimmune haemolytic anaemia, thrombocytopenia, polyarthritis, hepatitis and nephritis, the patient presented with life threatening hyperreactivity to viral infections as well as recurrent bacterial infections. This is in contrast to the very mild signs of skeletal dysplasia observed in this patient. Although short stature has been attributed to non-ossified lesions affecting the area of the growth plate the long bones and the spine [2], the growth retardation observed in our patient cannot be fully explained by the disturbances of bone growth, but may also be related to chronic consuming disease as well as nutritional factors.

Osteopontin functions as a major regulator of bone resorption in osteoclasts and stimulator of interferon-alpha production in plasmacytoid dendritic cells [6, 7]. Dephosphorylation of osteopontin by TRAP down regulates osteopontin activity. Indeed, patients with SPENCD were shown to exhibit higher levels of active osteopontin in serum, urine and dendritic cells [5]. Furthermore, TRAP-deficient dendritic cells secrete Th1-polarizing proinflammatory cytokines such as TNF-α [5]. In addition, a constitutive up-regulation of interferon-regulated genes was demonstrated in patients with SPENCD [4]. Consistent with chronic activation of the innate immune system, we demonstrate increased levels of type I interferon in CSF and serum as well as overexpression of interferon-stimulated genes in peripheral blood cells of the patient. Type I interferons have pronounced immunostimulatory effects and play a central role in antiviral defense. Indeed, chronic overproduction of type I interferons may have led to the hyperreactive inflammatory responses caused by viral infections in the patient. In addition, type I interferons play a central role in SLE pathogenesis by promoting the release of nuclear antigens from dying cells and maturation of autoreactive B-cells thereby inducing autoimmunity [8]. Interestingly, reduced level of TRAP and an interferon-signature is also observed in the clinically healthy parents. Of note, heterozygous mutations in *TREX1* and the *RNASEH2* genes, which cause the type I interferonopathy Aicardi-Goutières syndrome, confer an increased risk for SLE [9, 10] raising the question as to whether constitutive activation of antiviral immunity in parents of patients with SPENCD may also increase the risk for autoimmune disease later in life.

Type I interferonopathies such as Aicardi-Goutières syndrome and SPENCD constitute an emerging group of genetically determined diseases that are characterized by overproduction of antiviral type I interferon [11]. Although clinically distinct and genetically heterogeneous, these disorders share some phenotypic features such as neurological impairment including brain calcifications, multisystem inflammation and autoimmunity. Currently, there is no specific treatment available for these patients. However, our knowledge on the underlying pathomechanims suggests that patients with SPENCD may therapeutically benefit from inhibition of type I interferon signalling.

## Conclusion

SPENCD is a type I interferonopathy that may present with absent or very discrete signs of skeletal dysplasia. SPENCD should be considered in an infant or young child presenting with recurrent systemic inflammation, particularly if triggered by viral infections, immunodeficiency, autoimmunity and developmental delay even in the absence of metaphyseal dysplasia. Heterozygous parents of SPENCD patients exhibit an interferon signature in blood and may therefore be at increased risk for autoimmune disease.

## Consent

The study was conducted with approval by the ethics committee of the Medical Faculty, TU Dresden, and written informed consent was obtained from the patient’s parents.

## Abbreviations

AGS: Aicardi Gutieres syndrome
ACP5: Acid phosphatase 5, tartrate-resistant
ANA: Antinuclear antibodies
CSF: Cerebrospinal fluid
ESBL: Extended spectrum beta lactamase
TRAP: Tartrate-resistant acid phosphatase
